# Azithromycin does not improve disease severity in acute experimental pancreatitis

**DOI:** 10.1371/journal.pone.0216614

**Published:** 2019-05-10

**Authors:** Sebastian Weis, Mario Heindl, Tania Carvalho, Elisa Jentho, Jana Lorenz, Ines Sommerer, Joachim Mössner, Albrecht Hoffmeister

**Affiliations:** 1 Department of Anesthesiology and Intensive Care Medicine, Jena University Hospital, Jena, Germany; 2 Institute for Infectious Disease and Infection Control, Jena University Hospital, Jena, Germany; 3 Division of Gastroenterology, University Hospital Leipzig, Leipzig, Germany; 4 Instituto de Medicina Molecular, Faculdade de Medicina, Universidade de Lisboa, Lisbon, Portugal; Medizinische Fakultat der RWTH Aachen, GERMANY

## Abstract

Acute pancreatitis is a severe systemic disease triggered by a sterile inflammation and initial local tissue damage of the pancreas. Immune cells infiltrating into the pancreas are main mediators of acute pancreatitis pathogenesis. In addition to their antimicrobial potency, macrolides possess anti-inflammatory and immunomodulatory properties which are routinely used in patients with chronic airway infections and might also beneficial in the treatment of acute lung injury. We here tested the hypothesis that the macrolide antibiotic azithromycin can improve the course of acute experimental pancreatitis via ameliorating the damage imposed by sterile inflammation, and could be used as a disease specific therapy. However, our data show that azithromycin does not have influence on caerulein induced acute pancreatitis in terms of reduction of organ damage, and disease severity. Furthermore Infiltration of immune cells into the pancreas or the lungs was not attenuated by azithromycin as compared to controls or ampicillin treated animals with acute experimental pancreatitis. We conclude that in the chosen model, azithromycin does not have any beneficial effects and that its immunomodulatory properties cannot be used to decrease disease severity in the model of caerulein-induced pancreatitis in mice.

## Introduction

Acute pancreatitis (AP) is a severe systemic inflammatory disease that is mainly caused by gallstones and excessive alcohol consumption [1, 2]. Most of the cases of AP are mild and have non-fatal courses [3, 4]. However, for yet unidentified reasons, approximately one third of AP patients will develop a severe disease with increased morbidity and mortality [5, 6]. Since there is no causative therapy for AP, treatment of patients relies on fluid administration, analgesia, nutritional, and intensive care support [3, 7].

The exact pathophysiological events that occur during the development of AP are only partially understood. It is currently believed that initial autodigestion of the pancreas initiates a local and subsequently systemic inflammatory response syndrome (SIRS) that is shaped by bacterial translocation from the intestines [8] and to which extravasation of granulocytes and T-cell activation contribute fundamentally [9–11]. Due to the compensatory anti-inflammatory response syndrome (CARS) patients are at risk to immunosuppression predisposing the organism to infection [12]. Therapeutic strategies are needed to prevent the immunological response of the organism and thus prevent the progression of the disease to severe pancreatitis.

Antibiotic therapy is reserved for AP patients with infection of organ necrosis or fluid collections. Even in cases of necrotizing pancreatitis, the guidelines by the *American Gastroenterological Association Institute* do not recommend the usage of prophylactic antibiotics [3].

Macrolide antibiotics, such as azithromycin, clarithromycin or erythromycin, are bacteriostatic agents acting via a reversibly binding to the 50S subunit of the bacterial ribosomes. Thus they inhibit bacterial protein biosynthesis [13]. Experimental and clinical evidence shows that macrolides also possess potent anti-inflammatory and immunomodulatory properties [14–16]. As a consequence, neutrophil recruitment [17] or direct cytokine release by monocytes can be inhibited [18, 19]. In animal models of acute lung injury and inflammation macrolide therapy improves disease severity [20–22]. These alternative non-antimicrobial properties are used in patients with chronic pulmonary disease reducing the number of acute exacerbations [14, 23, 24]. In a recent retrospective study, macrolides also improved disease course in patients with acute respiratory distress syndrome [25]. Whether administration of macrolides is beneficial in AP has not been investigated so far. This might be due to the anecdotal report of AP as a complication of macrolide therapy [26]. In addition, early reports show that macrolides can decrease oxidative damage to endothelial cells independently of their anti-bacterial activity [27]. Hence an additional way to improve disease severity in acute pancreatitis might be via enhancing stress resistance of pancreatic tissue, i.e. via providing tissue damage control [28].

We here asked whether administration of the macrolide antibiotic azithromycin improves the course of acute experimental pancreatitis and could serve as a potential therapeutic agent via its anti-inflammatory properties.

## Materials and methods

### Animal experiments and induction of acute pancreatitis

Animals were kept under specific pathogen free conditions with an underlying 12 h day/night rhythm and fed with standard rodent chow and water. For all experiments, female C57Bl/6JRj mice aged 10 to 12 weeks were used. All animal experiments were performed according to the Guide for the Care and Use of Laboratory Animals published by the US National Institutes of Health (NIH Publication No. 85–23, revised 1996) and approved by the Landesdirektion Sachsen, the local authority for animal care (animal experiment registration number TVV 12/13). The animals were inspected and weighed every day, weight loss of 20% of the initial weight for more than two days was fixed as termination criterion. Criteria for the assessment of animal health and for discontinuation of mouse experiments were defined in accordance with the local authority for animal care and determined daily using a score sheet (S1 and S2 Files). Collecting of blood samples and sacrificing was performed under ether sedation.

AP was induced using an adapted mouse model of caerulein-induced severe acute pancreatitis as previously described by Zhang *et*. *al*. [29]. Prior to caerulein injection, mice were fasted for 18 h with free access to water. AP was induced by eight hourly intraperitoneal (i.p.) injections of caerulein (50 μg/kg body weight, Sigma) for three days. Control animals received 0,9% sterile saline at the same time points. During the phase of pancreatitis induction, access to food was omitted. One hour before the first caerulein injection mice were treated with azithromycin (*i*.*p*. 200 mg/kg body weight, Pfizer) or ampicillin (*i*.*p*. 30 mg/kg body weight or sterile phosphate-buffered saline as vehicle control (100 μL/10 g body weight) once a day for a total of three days.

Mice were treated with PBS, caerulein, azithromycin or ampicillin in parallel in six independent experiments.

### Serology

Whole blood was obtained by retroorbital bleeding under ether anesthesia and used for analyses. Plasma values were performed in mice-adjusted instrument settings at the Institute of Laboratory Medicine, Universitätsklinik Leipzig. Plasma was extracted from heparinized whole blood after centrifugation at 4000 rpm for 5 min. Measurement of plasmatic amylase and lipase was at the Institute of Laboratory Medicine, Clinical Chemistry and Molecular Diagnostics, of University Hospital Leipzig.

### Hematoxylin & eosin staining and immunohistochemistry

Mice were sacrificed by cervical dislocation, necropsies were performed and pancreas and lung were collected, fixed in 4% paraformaldehyde (OttoFischerGmbH, Saarbruecken, Germany) overnight and embedded in paraffin (Medite, Burgdorf, Germany). Samples from all animals were cut (5μm; Microm HM355S, Thermo Scientific, Walldorf, Germany), mounted and dried overnight at 45°C. Sections were stained with hematoxylin and eosin (HE) using a full-automatic microscope slide appliance (Shandon Varistain 24–4, Thermo Shandon Ltd.) as described [30]. Immunohistochemistry was performed as previously described [31]. A polyclonal rabbit antibody against myeloperoxidase was purchased from Abcam (Cambridge, UK) and used in a dilution of 1:100 in 2% donkey serum (SIGMA, St. Louis, USA). Peroxidase-labeled secondary antibody was purchased from Dianova (Hamburg, Germany). For visualization Liquid DAB^+^ substrate chromogen (Dako, Glostrup, DK) was applied. Counterstaining was done with hematoxylin. A monoclonal rabbit antibody against CD3 was purchased from Abcam (Cambridge, UK). For detection of B220 a purified rat anti-mouse CD45R (BD Biosciences) was used. H&E images were recorded with a Moticam 2500 mounted on a Zeiss AxioLab. Bright field images of the IHC-stained pancreatic and lung tissues were recorded on a BZ-8000 microscope (Keyence, Osaka, Japan) using a PlanApo objective (20x/0.75; Nikon, Melville, USA) at 200x magnification. The number of neutrophils was determined manually using Photoshop CS3 software (Adobe, San Jose, USA) by counting MPO^+^ cells in eight randomly captured high power fields (200x) per mouse. Severity of AP and lung injury was scored by a pathologist blinded to experimental groups (T.C.). Pancreatic lesions (edema, inflammation, vacuolation and necrosis) were scored as previously described by Rongione et al. [32]. Severity of lung injury was determined with a semi-quantitative score by assessing the extent of edema, hemorrhage, alveolar septum cellularity and perivascular inflammatory cell infiltration, according to a 4-tier score: minimal = 1; mild = 2; moderate = 3; marked = 4.

### Statistical analysis

Mann-Whitney-U and Kruskal-Wallis tests were applied for testing of differences between groups (Prism 5, GraphPad, San Diego, USA). Statistical significance was considered if p<0.05 and given when appropriate.

## Results and discussion

### Treatment with azithromycin does not affect disease severity of acute-experimental pancreatitis

**Fig 1:**
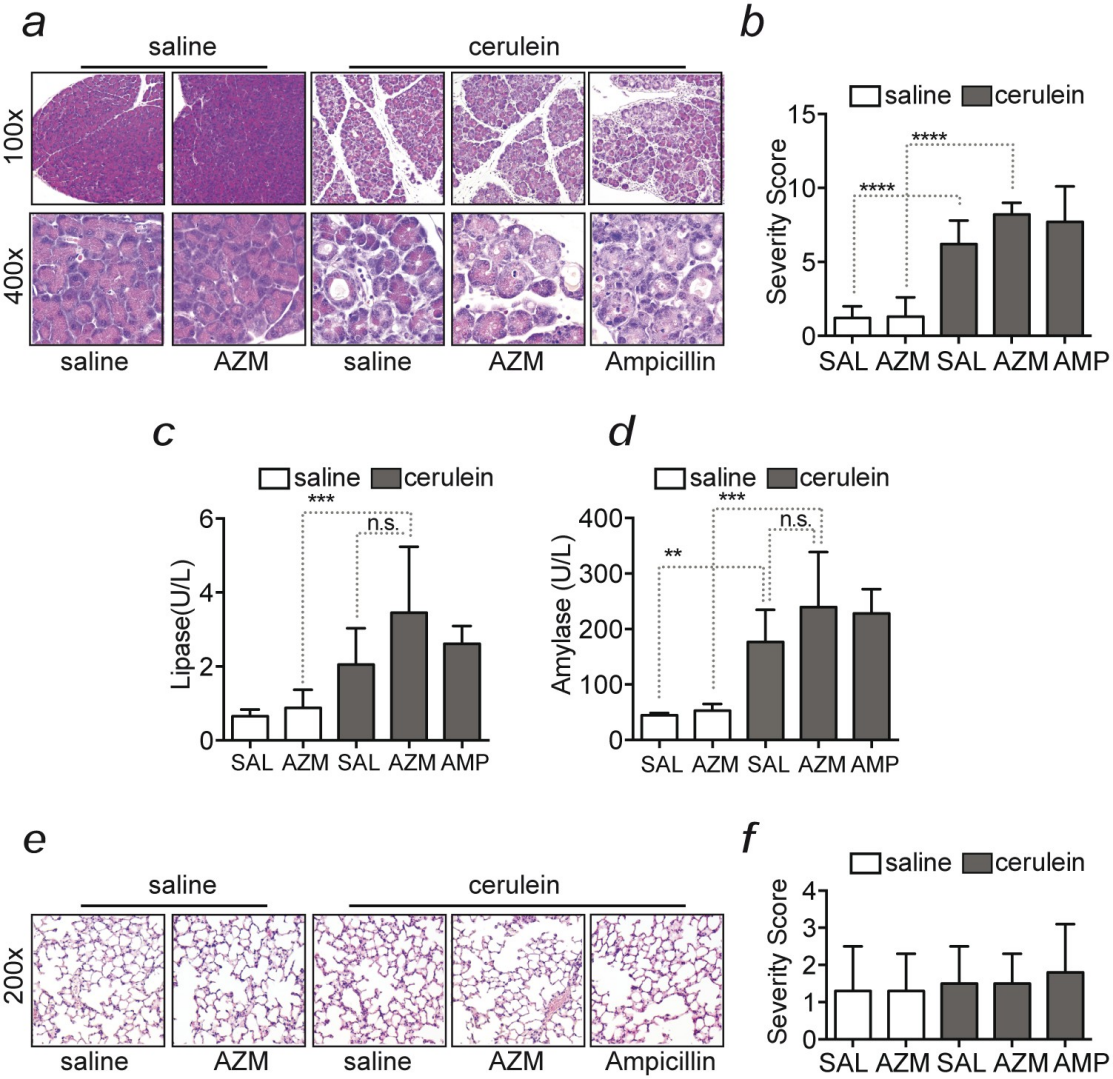
Azithromycin does not lessen disease severity of acute-experimental pancreatitis. **(a)** Representative images and (**b**) histopathological grading of pancreatic lesions 3 days after induction of AP or sham treatment of mice treated with azithromycin (AZM), ampicillin or saline. **(c)** Plasma amylase and **(d)** lipase at day three after AP induction. **(e)** Representative images of H&E stained lung sections of the same mice, and (**f)** histopathological grading of lung lesions (mean ± SD). Magnification as indicated 100x and 400x for pancreas and 200x for lung; H&E stained sections (a, e). Data is presented as mean±SD of six individual animals from six independent experiments. *p<0.5, **p<0.01, ***p<0.001, ****p<0.0001, n.s.: non significant. AZM: Azithromycin; SAL: saline.

All caerulein-treated mice developed pancreatitis of moderate severity, showing edema, inflammation, vacuolation and apoptosis of acinar cells, after three days of consecutive caerulein injection (Fig 1a and 1b). Plasma levels of amylase and lipase were significantly increased (p<0.01) at day three after AP initiation as compared to control animals that received saline or azithromycin only (Fig 1c and 1d). We could not observe differences between AP groups that were, in addition to caerulein, treated with saline, azithromycin or ampicillin neither in the histopathological severity nor in plasma levels of amylase and lipase (Fig 1a–1d). There were no lesions in the lung (Fig 1e–1f), despite that the used caerulein model has previously been described as bearing the features of AP associated acute lung injury [33]. This might be due to the adapted 3-day protocol in which early pulmonary damage might have already resolved. Overall, there were no differences in the severity of AP between mice that were treated with azithromycin and the mice that received saline or ampicillin (Fig 1).

### Azithromycin does not influence immune cell invasion in the pancreas during experimental AP

**Fig 2:**
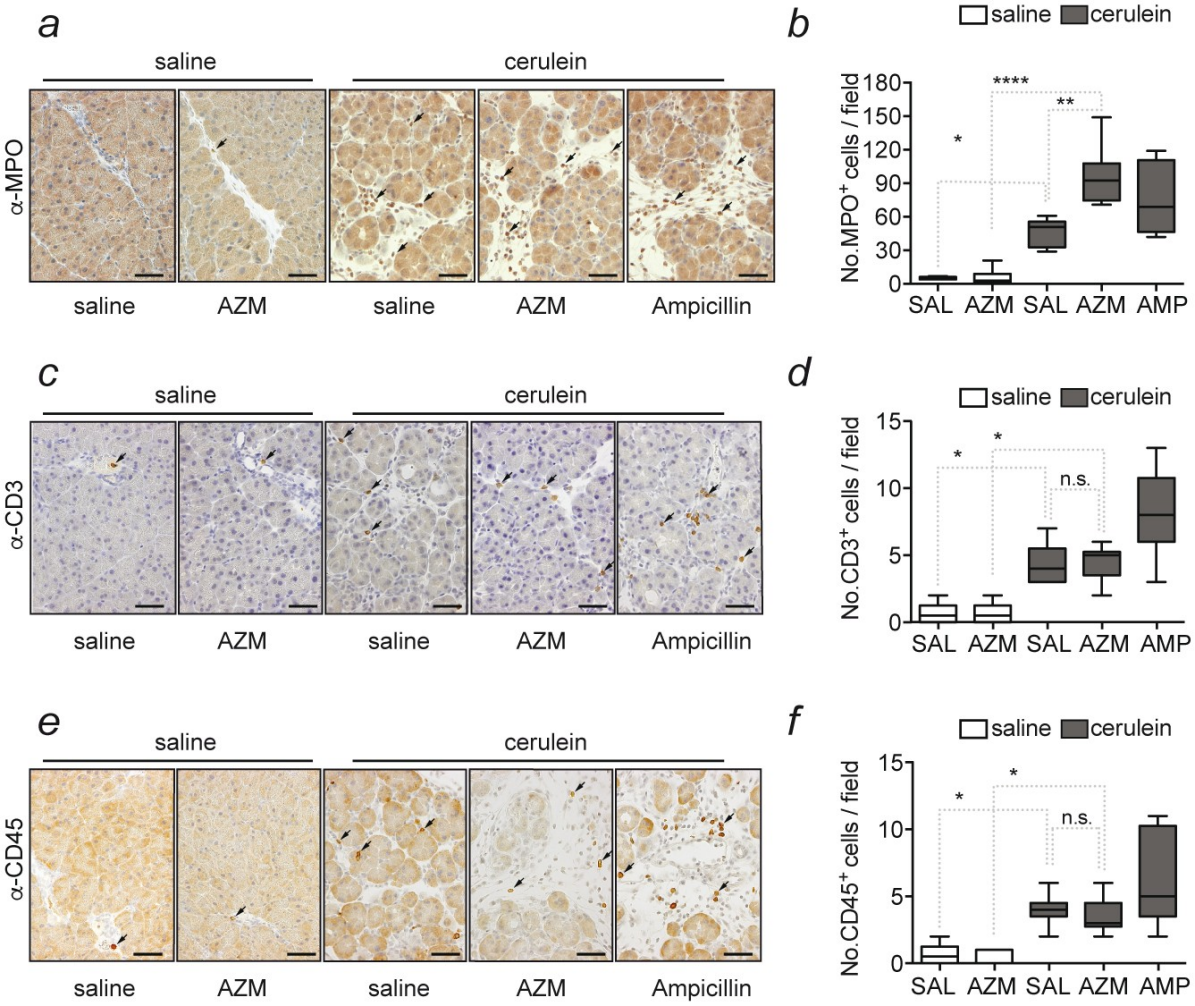
Immunohistochemical detection of myeloperoxidase, CD3 and CD45 in the pancreas during experimental AP. **(a)** Immunohistochemistry for myeloperoxidase (MPO) in the pancreas after induction of acute pancreatitis and treatment with saline, azithromycin (AZM) or ampicillin (AMP). Black arrows indicate MPO-positive inflammatory cells. **(b)** Quantification of MPO positive cells per field (200x) in mice receiving saline or caerulein and treated with either saline, AZM or ampicillin. **(c)** Immunohistochemistry for CD3. Black arrows indicate CD3-positive inflammatory cells within the pancreatic tissue. **(d)** CD3-positive cell counts per field. **(e)** Immunohistochemistry for CD45 **(f)** CD45 positive cell counts per field. DAB counterstained with hematoxylin; 200x magnification, scale 50 μm (a, c, e). Data is shown as Whisker plots with indicated means of six individual animals from six independent experiments. n.s. = not significant. *p<0.05. n.s.: non-significant. AZM: Azithromycin; SAL: saline; AMP: ampicillin.

In contrast to other experimental models, caerulein injection does not cause necrotizing pancreatitis [34]. The advantage however is that it might mimic the systemic inflammation that occurs in response to the initial sterile inflammation caused by autodigestion and acinar cell destruction [33]. Neutrophil granulocytes are the main early inflammatory cell population seen in the pancreas during caerulein-pancreatitis [9]. Even though there was no effect on severity of pancreatitis after azithromycin application, we asked whether at least the infiltration of the pancreas by myeloid cells was altered. We therefore performed immunohistochemistry for myeloperoxidase (MPO) of the pancreas. After caerulein injection, mice showed significantly increased number of MPO-positive cells infiltrating the pancreas as compared to saline injected control animals (p<0.05)(Fig 2a and 2b). Yet, there was no significant decrease of MPO-positive cells in animals treated with azithromycin as compared to saline or ampicillin treated mice. In contrast, we detected a significant increased number of MPO-positive cells (p<0.01) infiltrating the pancreas of azithromycin treated animals (Fig 2a and 2b). We also investigated whether infiltration of T-cells assessed by CD3 staining and B-cells assessed by CD45R staining, was affected by azithromycin administration. While there was a significant increase of both immune cell populations in AP mice as compared to control animals, no difference in AP pancreas in response to azithromycin was detected (Fig 2c and 2d). This indicates that azithromycin does not impair immune cell recruitment into the pancreas in acute caerulein-induced pancreatitis in mice.

### Reduced numbers of CD3+ cells in the lung during treatment with azithromycin

**Fig 3:**
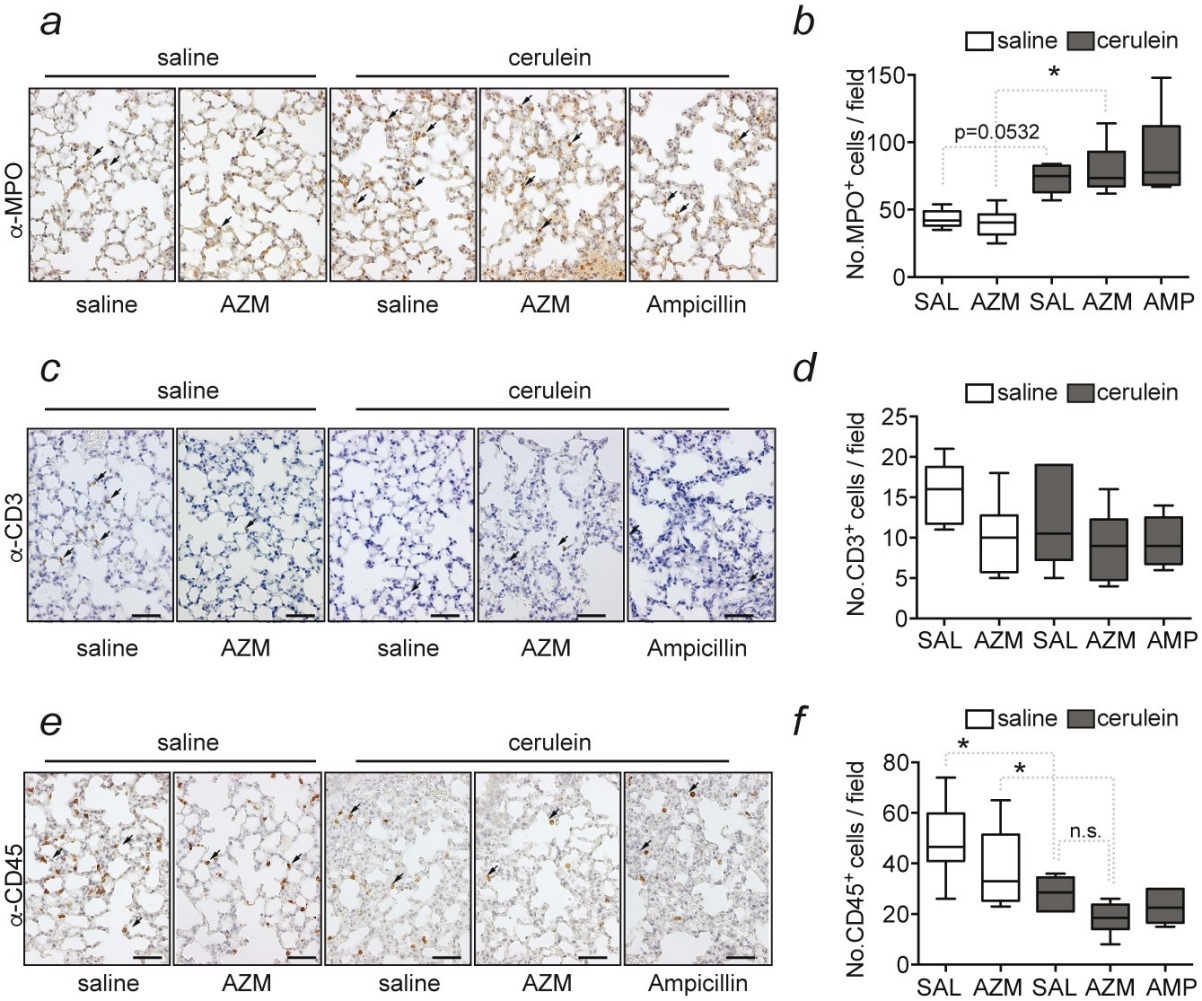
Immunohistochemical detection and quantification of myeloperoxidase, CD3 and CD45 positive cells in the lung during experimental AP. **(a)** Immunohistochemistry for myeloperoxidase (MPO) in the lung after induction of acute pancreatitis and treatment with saline, azithromycin (AZM) or ampicillin (AMP). **(b)** Corresponding quantification of MPO+ positive cell. **(c)** Immunohistochemistry of CD3 in lungs of mice subjected to pancreatitis and treatment with saline, azithromycin (AZM) or ampicillin (AMP). (**d)** Corresponding quantification of CD3+ positive cells. **(e)** Immunohistochemical staining for CD45. **(f)** Corresponding quantification of CD45+ positive cells. Representative images are shown in 200x magnification. Scale bar represents 50 μm in all images. Data is shown as Whisker plots with indicated means of six individual animals from six independent experiments. n.s. = not significant. *p<0.05, n.s.: not significant. AZM: Azithromycin; SAL: saline; AMP: ampicillin; AZM: Azithromycin; SAL: saline; AMP: ampicillin.

With regards to lung injury, which is commonly reported in AP, no histological changes were seen in this organ in any of the mice analyzed (Fig 1c and 1d). Nonetheless, we wondered whether the lung could also be targeted by inflammatory cell infiltration, similarly, to what we observed in the pancreas. Lung sections were immunostained for MPO, CD3 or CD45R (Fig 3), and while there were increased numbers of MPO-positive neutrophils in lung sections of the caerulein-treated groups when compared to saline controls, infiltration of MPO positive cells was not significantly altered by azithromycin treatment (Fig 3a and 3b). Similar to the results seen for the pancreas, immunostaining for CD3 and CD45R were performed in the lung of all mice to quantify lymphocyte infiltration. Among the saline controls a significant reduction of CD3-positive cells was observed in mice that received azithromycin (Fig 3c and 3d). In contrast, the number of CD45R-positive cells did not differ between groups (Fig 3e and 3f).

In summary, we cannot provide evidence that azithromycin affects lymphocyte or neutrophil infiltration or ameliorates tissue damage in acute experimental pancreatitis in mice. The immunomodulatory azithromycin does not seem to be a therapeutically relevant agent in this setting. In how this holds true for other non-secretagogue models of acute pancreatitis that are associated with a more severe disease course remains to be established. It is possible that the underlying initial sterile inflammation is not affected with azithromycin and that repair processes as mediated by macrophages would be enhanced. Yet, this model does not allow to investigate pancreatic repair due to its mild course. Also, we have not measured azithromycin concentrations in the pancreas. On the other hand, it has been shown that short-term application of azithromycin enhances the oxidative burst of neutrophils in patients [35]. It can be speculated that this initial burst increases AP disease severity, which is balanced by an improved immune response, hence with no net beneficial effects.

## Supporting information

S1 File Beispiel score sheet 3d Modell. Used score sheet with criteria for the assessment of animal health and for discontinuation of mouse experiments in German.
(PDF)

S2 File Translation of the score sheet for the assessment of animal health (S1 File) in English. (DOCX)
