# Supplementary material for: Azithromycin does not improve disease severity in acute experimental pancreatitis
Source: PLoS One. 2019 May 10;14(5):e0216614. doi: 10.1371/journal.pone.0216614 (PMC6510415; doi:10.1371/journal.pone.0216614)
Supplement: S1 File — Used score sheet with criteria for the assessment of animal health and for discontinuation of mouse experiments in German. (PDF) [file pone.0216614.s001.pdf]

Anlage 2 zum Genehmigungsantrag

Tier No: M 139	Tag des Versuchsbeginns: 26.8.14		Tierversuch No: TV 12/13
**Gewicht vor Versuchsbeginn:**	19,0 g		

**Datum**	25.8.14	26.8.14	27.08.14	28.8.14
**Versuchstag**	-1	0	1	2
**Uhrzeit**	14⁰⁰	7⁰⁰	7⁰⁰	7⁰⁰
**Inspektion**	14⁰⁰	* 7⁰⁰	* 7⁰⁰	* 7⁰⁰
Nahrung (**W**asser, **T**rockenfutter)	W T Nahrungsentzug	ab 9⁰⁰ Futter		
inaktiv	-			
isoliert	-			
Beine, Bauchhaut bläulich	-			
Hautwunden				
Aszites				
Abgebissene Zehen				
Atmung (**N**ormal, **F**lach, **S**chnell)				
Zittern				
Krämpfe				
**Pflege/Manipulation**				
Keine Nahrungsaufnahme				
Keine Flüssigkeitsaufnahme				
Vocalization on gentle palpation				
Menge getrunkenes Wasser (Durchschnitt pr Maus im Käfig) mL				
Gewicht in g		19,0 g	19,5 g	18,5 g
% Gewichtsveränderung				
Tier fühlt sich kalt an				
Injektion von Caerulein in mg/NaCl		8×50 µg/Kg/KW	8×50 µg/Kg/KW	8×50 µg/Kg/KW
Keine Auffälligkeiten				

**Andere:**	Bei mehr als einem klinischen Zeichen Leiter /stellvertretender Leiter des Versuchsvorhabens informiert	Tier getötet am: 28.8.14 17³⁰
**Unterschrift:**		

* ab 7⁰⁰ stündlich bis 16⁰⁰

N. Kolb
