# Supplementary material for: Azithromycin does not improve disease severity in acute experimental pancreatitis
Source: PLoS One. 2019 May 10;14(5):e0216614. doi: 10.1371/journal.pone.0216614 (PMC6510415; doi:10.1371/journal.pone.0216614)
Supplement: S2 File — (DOCX) [file pone.0216614.s002.docx]

| **Animal No:** | | | **Day of start of the experiment:** | | | **Animal experiment No:** | | | |
| --- | --- | --- | --- | --- | --- | --- | --- | --- | --- |
| **Weight before the start of the experiment:** | | | | | |  | | | |
| **Date** |  | | |  |  | |  | |  |
| **Test day** | **0** | | | **1** | **2** | | **3** | |  |
| **Time** |  | | |  |  | |  | |  |
| **Inspection** |  | | | | | | | | |
| Food (**W**ater, **T**: dry food) | W, T | | |  |  | |  | |  |
| inactive | - | | |  |  | |  | |  |
| isolated | - | | |  |  | |  | |  |
| Legs, abdominal skin bluish | - | | |  |  | |  | |  |
| Skin lesions, wounds |  | | |  |  | |  | |  |
| Ascites |  | | |  |  | |  | |  |
| Bitten phalanges |  | | |  |  | |  | |  |
| breathing (**N**: normal, F: shallow, S: rapid) |  | | |  |  | |  | |  |
| trembling |  | | |  |  | |  | |  |
| cramps |  | | |  |  | |  | |  |
| **care/manipulation** |  | | | | | | | | |
| Reduced food intake |  | | |  |  | |  | |  |
| Reduced fluid intake |  | | |  |  | |  | |  |
| Vocalization on gentle palpation |  | | |  |  | |  | |  |
| Amount of drunk water (average per mouse in the cage) mL |  | | |  |  | |  | |  |
| weight g |  | | |  |  | |  | |  |
| % weight change |  | | |  |  | |  | |  |
| Animal feels cold |  | | |  |  | |  | |  |
| Injection of **A**zithromycin in mg/**N**aCl |  | | |  |  | |  | |  |
| Injection of Caerulein in mg/NaCl |  | | |  |  | |  | |  |
| No abnormalities |  | | |  |  | |  | |  |
| **Other:** | | If there is more than one clinical sign, inform the leader / deputy head of the project | | | | | | Animal sacrificed on (date): |  |
| **Signature:** | | | | | | | | | |
